# Highly pathogenic avian influenza H5N1 virus infection of companion animals

**DOI:** 10.1080/21505594.2023.2289780

**Published:** 2023-12-08

**Authors:** Hinh Ly

**Affiliations:** Department of Veterinary & Biomedical Sciences, College of Veterinary Medicine, University of Minnesota, Twin Cities, MN, USA

**Keywords:** Influenza virus, flu, HPAI H5N1, dogs, cats, avian

While it is known that influenza viruses can frequently infect a variety of animal species, including humans (for a review, see ref. [1]), it is relatively uncommon for companion animals (e.g. pet cats and dogs) to be infected by the highly pathogenic avian influenza (HPAI) H5N1 virus. This article summarizes our current understanding of HPAI H5N1 infections in pets with the aim of raising awareness about these emerging and re-emerging infections in domestic animals.

Influenza viruses belong to the *Orthomyxoviridae* family and consist of four major types: influenza A, B, C, and D. Influenza A virus (IAV) can infect a wide range of avian and mammalian species, including some avian species (e.g. birds, ducks, chickens, turkeys), swine (pigs), equine (horses), and canine (dogs) species, besides humans. Influenza B virus (IBV) is known to infect both humans and seals, while influenza C virus (ICV) can infect humans and pigs. Additionally, recent evidence suggests that swine, cattle, and potentially humans [2] can also be infected by influenza D virus (IDV) [3]. However, the most significant human IAVs can cause annual (seasonal) epidemics and occasional outbreaks. These are new human IAV strains that can arise from an animal origin [via zoonotic transmission event(s)] and then spread rapidly among human populations with no pre-existing immunity against these new strains, which can result in excessive mortality and morbidity. There are four influenza pandemics in modern times, including the 1918–1919 Spanish flu, 1957 Asia flu, 1968 Hong Kong flu, and 2009 Swine flu (Figure 1). For example, the 1918–1919 Spanish flu caused the most damage, with an estimated 500 million infections and 50–100 million deaths worldwide [4].

**Figure 1.**
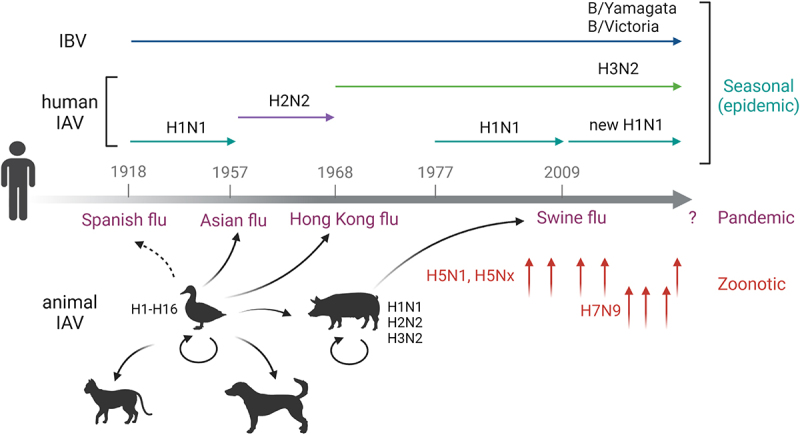
Influenza a (IAV) and influenza B (IBV) virus infections of humans and animals (e.g. avian, swine, canine, feline, and other animal species) to cause seasonal (epidemic) human infections or occasional outbreaks (Spanish flu, Asian flu, Hong Kong flu, and swine flu) that were caused by the different subtypes of human IAVs. Almost all subtypes of IAV (H1-H16, N1-N9) have water birds as their natural hosts, with some IAVs (e.g. the highly pathogenic avian influenza virus HPAI H5N1) capable of infecting other animal species, such as large felids (tigers and leopards) and domestic dogs and cats, and farmed animals (chickens, turkeys, ducks, and mink) as well as humans with a high case fatality rate. Drawing adapted from ref. [1] with modifications using Biorender.

Zoonotic influenza infections usually occur through occasional viral transmission from either avian or swine species to humans (Figure 1), but these types of infections are usually self-limiting. However, certain types of IAVs, such as avian H5 and H7 viruses, are known to cause hundreds to thousands of infections, with a high case fatality rate (30–50%) in humans (for a review, see ref. [5]). Therefore, the possibility that some of these highly pathogenic avian (HPAI) viruses can gain a foothold in human populations to mediate efficient human-to-human transmissions and a potential pandemic poses a significant public health risk [6]. Notably, since the initial report of human fatalities caused by the HPAI H5N1 infections in Hong Kong in 1997, there have been at least 878 cases of human H5N1 infections worldwide, resulting in a mortality rate of 52.16% (https://www.who.int/publications/m/item/cumulative-number-of-confirmed-human-cases-for-avian-influenza-a(h5n1)-reported-to-who– 2003–2023–14 July 2023). However, it is relatively uncommon to learn about HPAI H5N1 infections of other animal species besides known human cases and, of course, their natural avian host species until recent years. Since 2021, at least 12 different animal species, including some companion animals, such as household cats and dogs, have been reported to be infected by the HPAI virus H5N1 in more than nine countries (https://www.who.int/news/item/12-07-202312 July 2023-ongoing-avian-influenza-outbreaks-in-animals-pose-risk-to-humans).

A recently reported case of a domestic cat infected by the HPAI H5N1 virus (clade 2.3.4.4b) occurred in France in December 2022 [7]. This case involved a cat that lived with a human family next to a breeding duck farm. After the HPAI H5N1 clade 2.3.4.4b virus was confirmed in some ducks at the farm, more than 8,000 ducks were culled. Approximately a week later, the cat began to show signs of apathy and mild hyperthermia. As the cat’s condition worsened, with significant neurological and respiratory (dyspnoea) symptoms, it was compassionately euthanized by a veterinarian. The attending veterinarians collected sinonasal, tracheal, and anal swab samples after the animal’s death for screening by real-time reverse transcription PCR (RT-PCR) with primers targeting the matrix (M) protein and haemagglutinin (H5) genes, and the positive tracheal and sinonasal swab samples were sent to the National Reference Laboratory, which confirmed HPAI H5N1 virus by specific real-time RT-PCR assay for the H5 clade 2.3.4.4b and neuraminidase (N1) genes.

Phylogenetic analyses of HPAI H5N1 genomes indicated that the virus from the cat belongs to the A/duck/Saratov/29–02/2021–like genotype, which has been the predominant virus genotype circulating in Europe, including France, since September 2022. The virus sequence from the infected cat was directly related to virus sequences identified in the same area in December 2022, but differed from the viruses isolated from the neighbouring duck farm by only two nucleotides (out of 13,507 total nucleotides sequenced), which resulted in an E627K mutation in the polymerase basic protein PB2 and an E26G mutation in nonstructural protein 2. The E627K mutation has previously been described as a major marker of influenza virus adaptation to mammalian hosts [1,8], whereas the E26G mutation has been shown to play a role in virus adaptation to temperature changes [9].

Additional cases of cat infection by the HPAI H5N1 virus (in 25 of 46 cats examined) were discovered from June to July 2023 in Poland [10]. Molecular analyses showed that the virus belongs to the same genotype (clade 2.3.4.4b) as H5N1 viruses, as shown in the French study [7], and that these viruses were known to circulate in wild birds and poultry in Poland in the past. Suspected cat samples were tested by real-time RT-PCR using primers targeting the M gene, and subtyping was performed using primers specific for the H5 and N1 genes. The samples (19 of the H5N1 virus-positive samples collected from 25 cats with the highest viral loads) were subjected to whole-genome sequencing. Consensus sequences were generated and compared with previously obtained HPAI H5N1 virus genomes from Poland as well as sequences from other European countries that were made available in the Global Initiative on Sharing All Influenza Data (GISAID.org). The HPAI H5N1 viruses from the Polish cats differed from each other by 1–12 nucleotides and 0–8 amino acids, and when comparing those sequences with those of the most closely related viruses from birds (A/white_stork/Poland/MB244/2023), they showed at least four nucleotide and three amino acid mutations in two of the viral tripartite polymerase proteins (PB1-P64S, PB2-N82T, and PB2-K526R), which might explain their increased adaptation to mammals [1,11–17]. Although these viruses appear to be very similar, indicating a potential common source of infection, the actual animal source has not yet been identified, even though the same mutation (PB2-E627K) found in cats was present in the H5N1 virus detected in the white stork (A/white_stork/Poland/MB244/2023) at the start of the study.

Another outbreak of HPAI H5N1 infection in companion animals in Europe was reported in Northern Italy [18]. Specifically, on 17 April 2023, increased mortality was recorded in a backyard poultry farm located in the province of Brescia (Italy). The farm was quarantined quickly, and the samples were tested for HPAI viruses. Tracheal and cloacal swabs from birds and nasal swabs from humans at the farm were analysed by a battery of real-time RT-PCR tests, using primers targeting the M gene, and positive samples were further analysed to detect for the H5 and H7 genomic sequences as well as for neuraminidase subtype and H5 pathotype and for the H5-specific multi-basic HP cleavage site. Tracheal swabs from virus-positive hens were determined to be HPAI H5N1 viruses, whereas cloacal swabs from farm-kept ducks and geese, and all nasal samples from tested humans were negative. Complete genome sequencing of a virus-positive sample was performed using next-generation sequencing, and the sequences were aligned against the most closely related sequences identified from a BLAST search in GISAID. The phylogenetic tree topology of the eight viral gene segments indicated that the virus belonged to the same HPAI H5N1 clade 2.3.4.4b, genotype BB (H5N1-A/Herring_gull/France/22P015977/2022-like), and clustered together with the H5N1 viruses collected from black-headed gulls (*Chroicocephalus ridibundus*) in Italy during the first months of 2023. Molecular analysis identified an unusual mutation in the PB2 protein, T271A, which was previously shown to be a marker of viral adaptation to mammalian species [1,19,20].

Serum samples from a cat and five dogs belonging to the same backyard poultry farm were analysed for antibodies against IAV nucleoprotein (NP), haemagglutinins H5, H7 and neuraminidases N1, N2, N3, and N7 using different serological assays. The presence and specificity of H5 antibodies were confirmed by the haemagglutination inhibition (HI) test and microneutralization (MN) assay using two different viruses [HPAI-H5N1 A/Gull/Italy/23VIR1310–12/2023 (Gull/23) and LPAI-H5N3 A/Mallard Duck/Netherlands/38/2008 (Mal/08)], which belong to the Goose/GD lineage clade 2.3.4.4b and the Eurasian lineage, respectively. This battery of serological tests detected antibodies against NP, H5, and N1 in all sera collected from dogs and cats at the two sampling times. To further evaluate the specificity of the HI and MN assays, the authors used archived sera from 10 dogs and 10 cats that were collected during routine diagnostic tests to screen for the H5N1 Gull/23 antigen, which all showed negative results, indicating new HPAI H5N1 infections in dogs and cats at the backyard poultry farm.

Epidemiological evidence revealed that the Eurasian strain of HPAI H5N1 came to North America’s shore in late 2021 via wild migratory birds, causing an outbreak that is still ongoing and affecting at least 60 million commercial and wild birds in the USA [21] (https://www.cdc.gov/flu/avianflu/avian-flu-summary.htm). In January 2023, a 2-year-old neutered domestic short-haired male cat was brought to a Northeast Nebraska veterinary clinic [22]. The cat was kept outdoors and had been missing for a few days before being found in an abnormal (anorectic) state. Three other free-roaming outdoor cats were present on the premises. One day after the examination, the cat had a series of five seizure episodes despite the administration of diazepam and was euthanized for humane reasons. About a day later, another cat from the same residence (an 8-month-old, spayed female, domestic long-haired cat) was taken to the same clinic and found to have mild hyperalbuminemia. Ten days later, the cat became laterally recumbent with continual tremors and was euthanized due to poor prognosis. Meanwhile, somewhere else in Nebraska, a 6-month-old, female, spayed, domestic short-haired cat was brought to a veterinary clinic in Central Nebraska (on April 2023) for difficulty ambulating, lethargy, and anorexia. This animal resided outdoors and was one of nine cats on the premises. The cat was euthanized for humane reasons due to poor prognosis. Tissues and swabs from all three cases were processed for quantitative real-time polymerase chain reaction (qRT-PCR) using primers specific for the M gene, and were determined to be HPAI H5N1, whereas they were not detected in three living cats on the same premises in Northeast Nebraska. Positive cat samples were sent to the USDA National Veterinary Services Laboratories (USDA NVSL) and confirmed as Eurasian HPAI H5N1 clade 2.3.4.4b. This study also reported a range of lesions in the organs of infected HPAI H5N1 cats and implicated virus-induced endothelial damage, vasculitis, and vascular thrombosis as important features of the disease pathogenesis [22]. The preponderance of evidence indicates that pet cats and dogs in both continents can be infected with this highly pathogenic avian influenza virus.

Cases of HPAI H5N1 virus infections in domestic cats in the USA, France, and Poland described above [7,10,22] as well as in farmed fur animals (mink) in Finland and Spain [23,24] showed overt clinical manifestations characterized by severe respiratory distress and neurological signs, but the affected pets (dogs and cats) reported from Italy [18] were completely asymptomatic, raising a real concern for the possibility of subclinical infections of HPAI H5N1 viruses in domestic animals that might live in close proximity and/or have had frequent and direct contact with humans, which could lead to inadvertent zoonotic transmissions. Since the infection and transmission routes of these highly pathogenic avian influenza viruses in some farmed animals (e.g. mink) and companion animals (pet cats and dogs) are still poorly understood, additional surveillance efforts and experimental research on HPAI H5N1 infection of susceptible animal species are needed to fully understand the ecology, possible transmission routes, and disease pathogenesis of these HPAI H5N1 viruses to prevent potential future outbreaks.

It is important to note that domestic animals, such as pet cats, in other parts of the world (e.g. Asia) have previously been shown to be naturally susceptible to HPAI H5N1 infections (for a review, see ref. [25]. Several years after the reported outbreak of HPAI H5N1 infections in humans in Hong Kong in 1997, this virus re-emerged in Asia, causing high mortality rates in poultry in addition to being responsible for acute respiratory infection in humans. Notably, the Asian HPAI H5N1 virus was also found to infect and kill mammals other than humans and birds. These animals included large cats (tigers), domestic cats, and other felids. Several outbreaks of fatal diseases caused by the Asian HPAI H5N1 viruses in large felids (tigers and leopards) and domestic dogs and cats have been reported in Thailand [26,27]. Therefore, if history is a guide, we can reasonably expect that the continuous evolution of HPAI H5N1 viruses through mutations, including antigenic shifts and drifts, will allow them to adapt to different animal hosts that include but are not necessarily limited to mammals (e.g. household pets) and to cross country boundaries or leap across vast distances between continents.

## Data Availability

No primary data is included in this article.
